# Donor-centric administration of the stool donor program is vital to its feasibility and patient safety

**DOI:** 10.1080/19490976.2025.2508950

**Published:** 2025-06-18

**Authors:** Amanda J. Kabage, Parker J. Haselhorst, Alexander Khoruts

**Affiliations:** Division of Gastroenterology, Hepatology, and Nutrition, Microbiota Therapeutics Program, University of Minnesota, Minneapolis, MN, USA

**Keywords:** Fecal microbiota transplantation, microbiota transplant therapy, stool donor, stool bank

## Abstract

Human stool-based products composed of fecal microbiota are a new frontier of medical therapeutics development. The development of standardized manufacturing protocols of donor microbiota has transformed fecal microbiota transplantation (FMT) from a crude and rarely used procedure to a widely accepted and highly effective option for treatment *Clostridioides difficile* infections. There is also a growing interest in using microbiota transplant therapies for multiple other clinical indications. In this manuscript, we review the logistical challenges experienced by various stool banks and our own group in establishing and administering a stool donor program. Furthermore, we explore and highlight the multiple ethical considerations that are ultimately essential to product safety and efficacy and propose basic principles that are necessary to maintain stool donor program integrity.

## Introduction

Over the past decade, fecal microbiota transplantation (FMT) became a widely accepted treatment for recurrent *Clostridioides difficile* infection (rCDI). The ready availability of FMT material to healthcare providers has been enabled by the development of standardized protocols for donor selection and cryopreservation of fecal filtrates,^1^ which facilitated the emergence of the ‘stool bank model’. Until recently, most FMT procedures in the US utilized cryopreserved fecal microbiota products distributed by OpenBiome, an international nonprofit stool bank.^2^ Similar stool bank operations have emerged globally, operating on more localized scales in regions such as the European Union, United Kingdom, Australia, and Canada, each adhering to distinct regulatory frameworks that are evolving within their respective countries.^3–11^

The stool bank model operates through a centralized system, which recruits and qualifies donors, manufactures and stores FMT products, distributes FMT to clinical providers, monitors safety and efficacy, and conducts pharmacovigilance. The model ensures rigorous donor screening and testing, facilitates access to cost-efficient treatments to rCDI patients, and enables large-scale safety reporting.^6,12,13^ In November of 2022, the US Food and Drug Administration (FDA) approved live-jslm (Rebyota, Ferring Pharmaceuticals) and simultaneously ended its ‘enforcement discretion policy’ that had until then permitted the operation of nonprofit stool banks.^14^ Nonetheless, the manufacturing of commercial FMT-based products still relies on the same basic ‘stool bank model’, which depends on human donors to provide the raw material for product manufacturing.

In this manuscript we review the reported experience of stool banks and describe the experience of our own Microbiota Therapeutics Program (MTP) at the University of Minnesota, which has been operating formally since 2013. One of the missions of MTP is manufacturing FMT-based products for management of patients with complex *C. difficile* infections being treated at the University of Minnesota Medical Center. In addition, between years 2022 and the end of 2024, MTP was also the manufacturer of FMT products that were distributed by OpenBiome for treatment of *C. difficile* infections under a brief extension of the FDA ‘enforcement discretion’ policy. However, the larger mission of MTP is in supporting multiple early phase research clinical trials for various indications across many academic institutions across the US. As some of these trials impose additional manufacturing constraints and requirements, the experience of the MTP donor program is instructive in anticipating the evolving expectations from stool bank operations.

While the dominant focus of prior literature describing the experience of establishing a large-scale stool bank has been on its feasibility and patient safety,^3–5,7,10,15–20^ little attention has been given to the ethical considerations pertaining to the critical role of human stool donors. Mikail et al. have previously drawn attention to the potential concerns and risks involved in being a stool donor.^21^ In this review we expand on some of the these points but also emphasize that donor experience is ultimately also vitally important to product safety for FMT recipients.

## Brief overview of the current MTP manufacturing workflow

**Figure 1.**
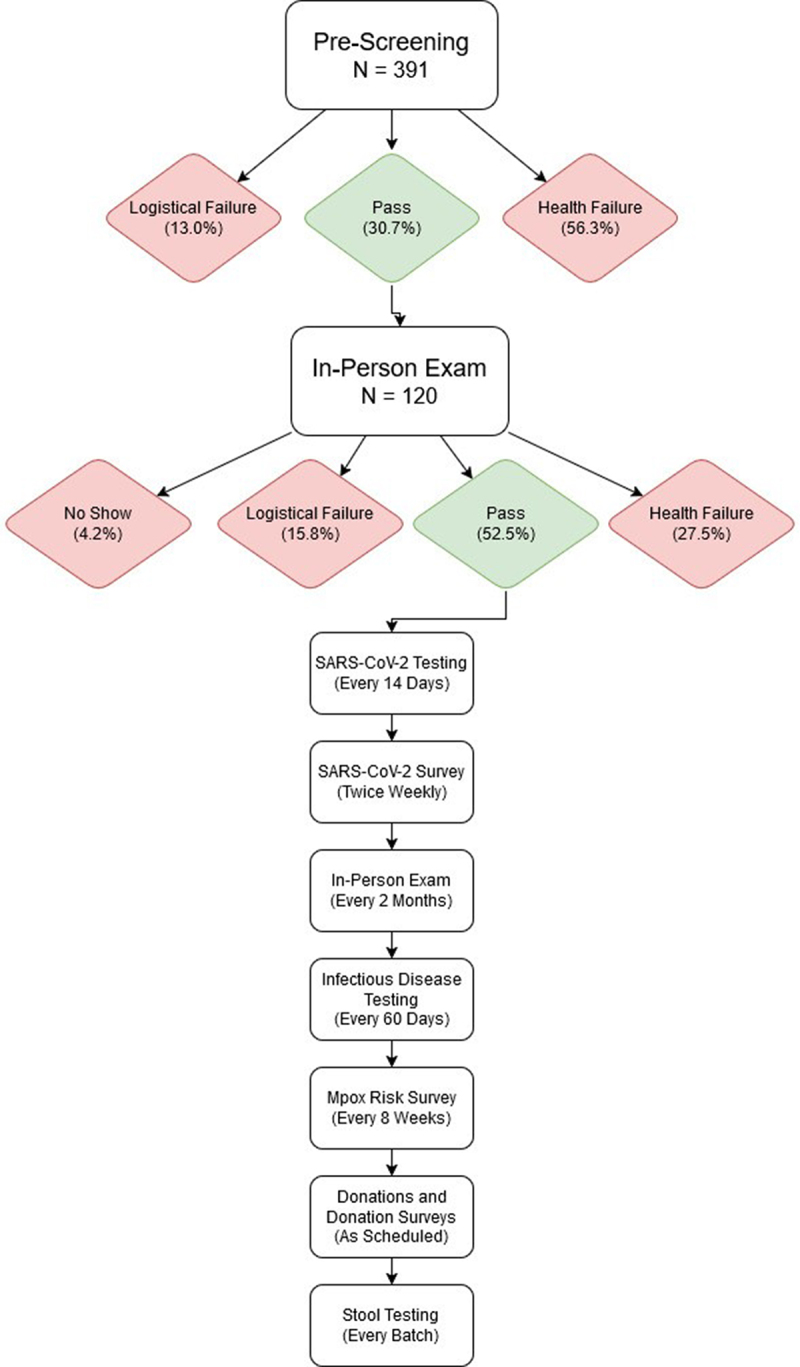
Flowchart of the MTP stool donor program screening and testing process.

All FMT product manufacturing by MTP is done in accordance with the FDA-approved protocols detailed in the Investigational New Drug Application (IND) and Good Manufacturing Practices (GMP). Human stool is the raw material for manufacturing. The basic workflow of donor screening and testing is outlined in Figure 1, and additional details are provided in the Supplementary Table S1. Qualification of the donors into the program follows satisfaction of inclusion and exclusion criteria. However, release of FMT products from quarantine follows a formal review by the Quality Assurance/Quality Control Department of the GMP facility of all clinical safety data and manufacturing batch records.

All stool donations are performed in a dedicated, supervised bathroom and transported under documented chain of custody to the GMP manufacturing facility on wet ice. The stool is processed within 4 hours of arrival to extract the microbial fraction, which is then frozen with a cryo- or lyoprotectant, depending on the final product formulation (liquid, capsules, powder). The manufacturing suite is allowed to process each donation from an individual donor one at a time, and the suite undergoes formal cleaning following each round of processing. These requirements prevent any potential for cross contamination of the product by microbes from a different donor. A sample of stool is retained from each donation for potential safety testing in the future in case of an adverse event.

The FDA requires full bookend infectious disease serology testing (at most 60 days apart) AND at least 15 days following the last donation. The donors are also required to undergo testing for SARS-CoV-2 at intervals no longer than 2-weeks and answer extensive questionnaires pertinent to infectious disease risk throughout their participation. The program excludes healthcare workers or caregivers in contact with patients or residents in long-term care facilities. Testing for enteric pathogen and multi-drug-resistant organisms (MDROs) is done on every batch of stool. In addition to infectious disease screening and testing, the donors undergo regular, detailed physical examinations and comprehensive laboratory evaluations to ensure their metabolic, immunologic, neurologic fitness, and psychiatric fitness.

Importantly, most research clinical trials supported by our program require repeated oral dosing of donor microbiota over weeks or months.^22,23^ Since the FDA does not allow mixing of donor stool, the recipients may receive FMT products manufactured from the stool of a single individual during their microbiota transplant therapy. This requirement also facilitates investigations into donor-associated variability in outcomes, which ultimately have the potential to inform rational donor selection strategies based on microbiome or metabolome-informed testing.^24^ From the manufacturing standpoint, this generally requires sustained and intensive commitment on the part of the donors over weeks or months.

## Emphasizing personal interactions and leveraging donor social networks enhance the efficiency of the recruitment process

The obvious initial challenge for any stool bank is donor recruitment. At first glance this might seem a trivial exercise, as all humans produce stool, and even imposing the most stringent health-based inclusion/exclusion criteria should still result in a pool of many thousands of potentially eligible individuals.

This naïve view was illustrated by the recent recruitment efforts by Ferring Pharmaceuticals. Its billboards and recruitment flyers blanketed entire residential neighborhoods of a large metropolitan area, sports stadiums, and the Twin Cities campus of the University of Minnesota. The poor yield of this approach was suggested by a surprisingly generous allowance for obese donors with a body mass index up to 35 kg/m^2^, which is contrary to consensus guidelines for donor selection,^25,26,27^ and a growing financial incentive. Early efforts by academic programs have proven to be similarly challenging. For example, Hota and colleagues in Toronto reported being able to retain only 2 individuals out of 322 initial responders following sequential screening steps that included website self-screening questionnaire, telephone interview, in-person examination, and microbiological testing.^3^ A similarly rigorous 3-stage screening process was adopted by the OpenBiome investigators, who reported that out of nearly 8,000 prospective donors, they were able to qualify 134 individuals (~1.7%) to be fully eligible to provide stool donations.^16^

In the earliest years of operation, MTP also used flyers in its attempts to recruit potential stool donors. The University of Minnesota campus with its 75,000 students, faculty, and staff seemed to have the ideal target population for recruitment. However, the approach was soon abandoned due to poor initial response rates and ~ 90% ineligibility among the responders. An early breakthrough was achieved after the Program Director gave a series of campus lectures about FMT, which were helpful especially as these took place at a time of little public awareness about the gut microbiome.^28^ Subsequently, a formal student group (*Microbiota in Health and Medicine*) was organized, which became involved in donor recruitment activities through direct engagement with student peers. Ultimately, as the Program matured, active stool donors became the most helpful recruiters through their own social networks (Table 1). Overall, the majority of our donors are either not affiliated with the University or work as staff or graduate students (Table 2).

**Table 1.** Recruitment methods and screening status.

How did you hear about the program?	Completed pre-screening	Failed pre-screening	Failed In-person Screening	passed all screening
ACT (MTP nonprofit partner)	4	3	0	1
Another Donor	52	30	6	16
Another Stool Bank	6	3	1	2
Friend or Family Member	49	32	9	8
Job Search – Money	3	3	NA	NA
MN State Fair	2	2	NA	NA
MTP Team Member	49	27	12	10
Media Story about FMT	7	6	0	1
Online Search	83	71	5	7
Recruitment Flyer	62	46	11	5
U of M Employee Referral	22	14	5	3
U of M Student Referral	43	28	7	8
U of M Website	7	4	1	2
Word of Mouth	2	2	NA	NA
**Total**	**391**	**271**	**57**	**63**

**Table 2.** University of Minnesota affiliation of stool donors and duration of participation in the MTP stool bank program.

Affiliation of stool donors	Total number of donors	Median duration in months (Range)
Undergraduate Student	9	5 (1, 87)
Graduate Student	18	11 (3, 44)
University Staff/Faculty	12	19 (7, 62)
No Affiliation with the University	24	11.5 (1, 86)

## Donor motivation is a key factor in donor retention and optimizing patient safety

Given the significant resources needed to establish the initial donor eligibility and the sustained commitment necessary to manufacture material from the same individual for many non-*C. difficile* indications, donor retention can be an existential issue. Thus, it is critical to understand donor motivation for participating in the program. Financial incentives provide one potential motivator. It is certainly prominently used by commercial manufacturers of FMT-based products, as illustrated on the Seres Therapeutics website.^29^ Ferring Pharmaceuticals flyers mentioned above promoted the potential for earning up to $1,600US per month, an offering that was further publicized on social media. An example of the response was illustrated by a Reddit user, who shared the flyer posted in a university bathroom with the caption: “It’s a dream come true fellas. You’ll now be getting paid to poop.”^30^ Clearly, financial motivation alone creates a substantial moral hazard and provides an incentive for donors to misrepresent their health. For example, in addition to not being truthful about infectious disease risk factors on health questionnaires, the individual may not admit to recent exposure to antibiotics.

Some European stool banks are building upon the existing blood banking infrastructure to recruit stool donors.^7,18^ Such institutions are not allowed to pay stool donors, similarly to the practice of not compensating blood donors in many countries. It is well established in blood banking that altruistically motivated donors tend to be more trustworthy and volunteer blood supply is safer for the patients.^31–33^ However, as noted above, participation in a stool donor program places much greater logistic and physical demands on the individual relative to blood donation. At the minimum, it is fair to compensate the donors for their time, inconvenience, and travel expenses. Hota et al. reported a nominal incentive of $150CA per 15 stool donations.^3^ Paramsothy et al. reported $20AUD reimbursement per donation without the requirement to donate on site. In the earliest days of our own program, we did not provide any compensation to our donors. This was feasible for the minimal clinical demands at the time, but we soon had to reevaluate our position. In 2013, we set up a focus group with active and past donors and asked, “what amount of compensation can be paid for a stool donation such that it does not become a financial inducement for participation?”. The group determined that amount to be $20US, which has been our compensation approved by the Institutional Review Board.

Chen et al. have studied different motivators of the stool donors providing stool to OpenBiome.^6^ They found that most of their donors (69%) were motivated by a combination of factors, including helping patients with C. *difficile* infections, supporting research, and earning money.^6^ A small fraction (12.5%) were motivated solely by their desire to help patients or research.^6^ However, the majority of donors did not last beyond 6 months, and the frequency of donations by purely altruistic donors was not reported. Notably, relatively short-term participation in the donor program may be acceptable if the transplant material is intended for treatment of *C. difficile* infections, where a single dose is sufficient to achieve cure. However, long-term donor retention becomes critical for manufacture of products intended for non-*C. difficile* indications, which involve much more intensive regimens of microbiota dosing, while the donor pool may be further constricted by additional selection criteria based on microbiome and/or metabolome profiling. Thus, approximately half of the donations in our program came from individuals who remained active in our program over 3 years (Table 3).

**Table 3.** Duration of participation and total stool donations.

Donation duration	Total donors	Donation totals
1–3 months	12	330
4–6 months	6	153
7–9 months	8	318
10–12 months	8	635
1–2 years	16	1,489
2–3 years	5	365
3–4 years	2	792
4–5 years	2	258
5–6 years	0	0
6–7 years	1	840
7–8 years	3	1,442
**Total**	**63**	**6,622**

A critical key to long-term donor retention is relationship building with the participants. Not surprisingly, the donors are gratified learning the stories of patients being helped with FMT. In our experience, most donors are keenly interested in microbiome research and the ongoing microbiota transplant clinical trials. The opportunities for these updates are provided during their regularly scheduled physical exams. In addition, we invite stool donors to attend our philanthropic fundraising events, where we review the different ongoing clinical trials being supported by FMT products manufactured in our program. Finally, we actively solicit feedback on the activities of the program to understand any elements in donor experience that we can improve.

## Direct stool testing strategy improves the detection of transient colonization by potential enteric pathogens

Over the years, one of the strategies for enteric pathogen testing allowed by the FDA and other regulators was 60-day bookend testing.^34^ We have chosen to perform direct testing for enteric pathogens and MDROs on every batch of donations collected within the 76-hour manufacturing window to ensure detection of transient colonization by undesirable organisms. In addition, to minimize the chance of processing infected stool, we perform pre-testing for enteric pathogens and MDROs during the week prior to scheduled donations.

The experience of this testing strategy showed that (1) transient passage or colonization by potential pathogens can be frequently detected in completely asymptomatic individuals, and (2) a 60-day bookend testing schedule can miss potential pathogen detection. From 2020 to 2024, we performed 489 stool tests for enteric pathogens and MDROs, of which 44 (9%) were positive (Supplementary Table S2). Some of these tests were repeats after an initial positive result on the same donor (14 tests). The majority of the donors (65%) had at least one positive stool test during their participation in the program. The remaining 35% were infrequent donors, averaging 24 donations over their history of their participation. Similarly to Rondinella et al.,^35^ we observe increased prevalence of asymptomatic carriage of potential pathogens in association with vacations and travel (data not shown). Of the 30 instances of positive stool testing, negative testing was documented in 22 instances within less than 60 days. Although our pretest strategy does reduce wasted manufacturing efforts, 36% of positive tests were obtained on direct testing of stool being processed despite a prior negative pretest. We found the average time between a positive test result and a negative test result to be 32 days. Therefore, we offer retesting at 4 weeks after a positive test unless there is another reason for the donor to be on hold. The relatively high detection rate of potential pathogens revealed by direct testing of every batch may also raise concerns about the strategy of mixing donations from multiple individuals into the same preparation. In the United States this practice is not allowed by the FDA. However, it has been used in other countries, especially for non-*C. difficile* indications.^36^

## Stool donors may experience health-related anxiety and require clear communication regarding their health status

As noted above, only a small percentage of individuals applying to the stool donor program will be ultimately qualify. Disqualification can result from the screening questionnaire or laboratory test results. However, most applicant potential donors consider themselves to be healthy. Without proper counseling, these individuals experience feelings of uncertainty and anxiety.^37^ Thus, our program has received multiple inquiries from healthcare providers seeking guidance on appropriate counseling information for individuals rejected from a local commercial stool donor program because of asymptomatic carriage of potential enteric pathogens. The question arises whether follow-up counseling should be offered by stool banks conducting donor testing.

Blood banks are responsible for timely donor notification, education, and guidance after abnormal laboratory results that have donor health or transmissibility implications.^38,39^ Stool donors will inevitably receive a positive laboratory test results at some point given sufficient time of participation. Some of the most common findings included detection of potential enteric pathogens, extended-spectrum beta-lactamases, and SARS-CoV-2. An antinuclear antibody (ANA) test was introduced into our routine serologic battery at the inception of the program as a potential predictor of autoimmunity risk.^40,41^ Unfortunately, ANA is commonly positive despite its relatively poor predictive value, but positive results can become a potential source of anxiety. All abnormal test results in our program are communicated by the donor program physician. Following counseling, when appropriate the individual may be given a medical referral or offered additional testing. In one instance, we performed an endoscopic work-up after blood was noted in the stool by the manufacturing team. In another example, we followed up on a false positive serologic test for syphilis in a long-standing donor. This individual had multiple previous negative tests and no behavioral risk factors. The donor program physician discussed the results with the individual and ordered secondary confirmatory treponemal tests, which were sent to two independent laboratories and established a negative result.

## Stool donors may experience psychological stress, fear, and guilt from the potential to hurt others

Participation in a stool donor program requires intensive and continuous monitoring with frequent questionnaires, including ones that accompany each donation. The questionnaires include highly personal information, such as sexual practices, travel history, dietary details, and alcohol consumption. In essence, donor monitoring is comparable to an overbearing surveillance program, which may impose on individual autonomy and affect their behavior.^21^ It creates the potential of donors feeling morally judged or pressured to refrain from behaviors perceived to be risky.^21^

Donor distress may also result from potential adverse events that could be linked to their donation. The first death directly linked to FMT in a clinical trial made headlines when a patient died from an extended-spectrum beta lactamase-producing *E. coli* that was traced back to a stool donor.^42^ Another patient became ill with the same infection, which was also linked to the same donor. Such news may cause stress, anxiety, or guilt among donors from the stool bank from which the infection was transmitted, although these emotions could be amplified even further in a patient-selected donor model.

## Conclusions

The US Food and Drug Administration has always emphasized the need for informed consent for recipients of FMT. The agency did so in its 2013 publication of the enforcement discretion policy,^43^ and later it even offered specific language for discussing risks associated with potential transmission of MDROs, mpox, and COVID. However, minimal attention has been given so far to the biomedical ethics pertaining to stool donors. The issue has only grown in its urgency since manufacturing of FMT-based products is now largely controlled by pharmaceutical companies, while in the US nonprofit stool banks like OpenBiome have ceased their operations.

It is important to recognize human stool donors as more than mere sources of biological material. FMT may be classified to be a drug, but ‘transplantation’ of human-derived cells into a patient is still fundamental to this treatment. The process of donor selection and screening, and the storage of data and stool samples should follow basic requirements as implemented for other transplantation treatments and allow transparency and biovigilance processes to assure and improve the safety of FMT. Donor trust is fundamental to the integrity of a stool donor program, more so than even for blood donations. Exposure to medications, especially antibiotics, and various lifestyle factors, e.g., alcohol consumption, can have profound effects on the gut microbiota, which can ultimately compromise product efficacy and safety. Therefore, appropriate motivation for joining and staying in the donor program is a critical factor that needs to be screened and cultivated. Not surprisingly, the most common question from the patients receiving FMT is ‘who are the donors?’. Patients are typically reassured knowing that the donors do not sell their stool and participate because they want to help.

Addressing donor motivation as well as the multiple unique risks associated with being a stool donor is critical to maintaining a sense of true partnership and collaboration. We suggest that stool donor programs should exist as nonprofit organizations, which are able to function in a donor-centric manner. They should be overseen by Institutional Review Boards, as is currently done in strictly academic programs. Such organizations should be able to cultivate long-term relationships with the donors and frequently revisit and address donor concerns. Stool donor programs, by virtue of assembling collections of retained stool samples and participating in various clinical trials may also be in an excellent position to facilitate microbiome research. Continued interventional trials using microbiota transplant therapies is likely to be a critical resource for development of next-generation live biotherapeutic products for multiple emerging indications.

## Supplementary Material

Supplemental Material

## Data Availability

The authors confirm that the data supporting the findings of this study are available within the article and its supplementary materials.
