# Supplementary material for: Donor-centric administration of the stool donor program is vital to its feasibility and patient safety
Source: Gut Microbes. 2025 Jun 18;17(1):2508950. doi: 10.1080/19490976.2025.2508950 (PMC12184138; doi:10.1080/19490976.2025.2508950)
Supplement: Supplemental Material [file KGMI_A_2508950_SM4188.zip › Supplementary Table 1 R2.docx]

**Donor Screening and Testing Procedures:**

**Supplementary Table 1.**

| **Screening Procedures** | **Testing Procedures** |
| --- | --- |
| Pre-screening comprehensive health and demographics questionnaires and logistics information prior to in-person visit | Bookend serologic testing for HIV, HAV, HBV, CMV, and EBV is done at no greater than 2-month intervals AND at least 15 days after the last donation |
| In-person physician-administered comprehensive history and physical exam at screening and every 6 months to rule out any infectious, metabolic, autoimmune, gastrointestinal, or neuropsychiatric disorder | Bookend serologic testing for syphilis is done at no greater than 2-month intervals AND at least 15 days after the last donation |
| Screening questionnaire and an abbreviated, physician-administered history and physical exam every 3 months | Metabolic and autoimmunity tests are done at screening and every 6 months (lipid panel, fasting glucose, high sensitivity CRP level, and liver function tests, fluorescent anti-nuclear antibody) |
| COVID-10-specific questionnaire twice weekly | COVID testing via nasopharyngeal swab at no greater than 14-day intervals |
| Monkeypox questionnaire every 8 weeks | NAAT-based enteric pathogen testing is done at the initial evaluation AND every batch of stool for: *Clostridium difficile* toxin B, *Escherichia coli* O157:H7, Shiga toxins, *Enteropathogenic E. coli (EPEC), Salmonella, Shigella, Yersinia, Campylobacter, Pleisomonas, and Vibrio*; *Giardia*, *Cryptosporidium, Cyclospora,* and Cystoisospora (previously *Isospora)*; Rotavirus, Norovirus I and II, and adenovirus. |
| Abbreviated questionnaire to screen for any symptoms of infection, gastrointestinal disturbance, or a change in the environment (e.g., travel, family illness) with every donation | Cultures using antibiotic-impregnated agar for presence of MDROs (MRSA, ESBL, CRE, and VRE) are done on every batch of stool |
| All stool donations are done in a supervised bathroom to ensure chain of custody | Pregnancy tests in female donors are done every 3 months |
