# Supplementary material for: Donor-centric administration of the stool donor program is vital to its feasibility and patient safety
Source: Gut Microbes. 2025 Jun 18;17(1):2508950. doi: 10.1080/19490976.2025.2508950 (PMC12184138; doi:10.1080/19490976.2025.2508950)
Supplement: Supplemental Material [file KGMI_A_2508950_SM4188.zip › Supplementary Table 2 R2.docx]

**Positive Asymptomatic Stool Tests in Donors**

**Supplementary Table 2.**

| **Positive Asymptomatic Stool Test** | **Days Between Positive and Negative Tests** | **Outcome** |
| --- | --- | --- |
| Enteropathogenic *E. coli* (EPEC) | 56 | Cleared and remained in the program. |
| Enteropathogenic *E. coli* (EPEC) | NA - Repeat testing | Cleared and remained in the program. |
| Enteropathogenic *E. coli* (EPEC) | NA - Repeat testing | Chronic positive stool tests. Donor was removed from the program. |
| Enteropathogenic *E. coli* (EPEC) | NA - Repeat testing | Chronic positive stool tests. Donor was removed from the program. |
| Enteropathogenic *E. coli* (EPEC) | NA - Repeat testing | Chronic positive stool tests. Donor was removed from the program. |
| Enteropathogenic *E. coli* (EPEC) | NA - Repeat testing | Chronic positive stool tests. Donor was removed from the program. |
| Enteropathogenic *E. coli* (EPEC) | NA - Repeat testing | Cleared and remained in the program. |
| Enteropathogenic *E. coli* (EPEC) | 57 | Cleared and remained in the program. |
| Enteropathogenic *E. coli* (EPEC) | 22 | Cleared and remained in the program. |
| Enteropathogenic *E. coli* (EPEC) | 17 | Cleared and remained in the program. |
| Enteropathogenic *E. coli* (EPEC) | 15 | Cleared and remained in the program. |
| Enteropathogenic *E. coli* (EPEC) | NA | Donor went on an unrelated hold. Stool testing has not resumed. |
| Enteropathogenic *E. coli* (EPEC) | 42 | Cleared and remained in the program. |
| Enteropathogenic *E. coli* (EPEC), ESBL | NA - Repeat testing | Donor went on an unrelated hold. Stool testing resumed after the hold. Donor cleared and remained in the program. |
| Enteropathogenic *E. coli* (EPEC), Norovirus | 37 | Cleared and remained in the program. |
| Extended-spectrum beta-lactamase (ESBL) | 93 | Donor went on an unrelated hold. Stool testing resumed after the hold. Donor cleared and remained in the program. |
| Extended-spectrum beta-lactamase (ESBL) | NA - Repeat testing | Chronic positive stool tests. Donor was removed from the program. |
| Extended-spectrum beta-lactamase (ESBL) | NA - Repeat testing | Chronic positive stool tests. Donor was removed from the program. |
| Extended-spectrum beta-lactamase (ESBL) | NA - Repeat testing | Chronic positive stool tests. Donor was removed from the program. |
| Extended-spectrum beta-lactamase (ESBL) | 42 | Cleared and remained in the program. |
| Extended-spectrum beta-lactamase (ESBL) | 67 | Cleared and remained in the program. |
| Extended-spectrum beta-lactamase (ESBL) | NA | Donor eligibility changed. Removed from the program before retesting. |
| *Yersinia enterocolititica* | 63 | Cleared and remained in the program. |
| *Yersinia enterocolititica* | NA - Repeat testing | Donor eligibility changed. Removed from the program before retesting. |
| *Yersinia enterocolititica* | 23 | Cleared and remained in the program. |
| *Yersinia enterocolititica* | 45 | Cleared and remained in the program. |
| *Yersinia enterocolititica* | NA - Repeat testing | Cleared and remained in the program. |
| *Yersinia enterocolititica* | 13 | Cleared and remained in the program. |
| *Yersinia enterocolititica* | NA - Repeat testing | Cleared and remained in the program. |
| *Campylobacter* | NA - Repeat testing | Cleared and remained in the program. |
| *Campylobacter* | 40 | Cleared and remained in the program. |
| *Campylobacter,* Norovirus | 29 | Cleared and remained in the program. |
| Sapovirus | 48 | Cleared and remained in the program. |
| Sapovirus | 22 | Cleared and remained in the program. |
| Sapovirus | 7 | Cleared and remained in the program. |
| *Cryptosporidium* | 12 | Cleared and remained in the program. |
| *Cryptosporidium* | 70 | Cleared and remained in the program. |
| Enterotoxogenic *E. coli* (ETEC) | 97 | Donor went on an unrelated hold. Stool testing resumed after the hold. Donor cleared and remained in the program. |
| Enterotoxogenic *E. coli* (ETEC) | 20 | Cleared and remained in the program. |
| Adenovirus | 119 | Donor went on an unrelated hold. Stool testing resumed after the hold. Donor cleared and remained in the program. |
| Norovirus | 21 | Cleared and remained in the program. |
| *E. coli*/Shigella - *Escherichia coli* 0157 | 15 | Cleared and remained in the program. |
| Enteroaggregative *E. coli* (EAEC) | 32 | Cleared and remained in the program. |
| *Fusarium* | 28 | Cleared and remained in the program. |
